# Lumican Peptides: Rational Design Targeting ALK5/TGFBRI

**DOI:** 10.1038/srep42057

**Published:** 2017-02-09

**Authors:** Tarsis Ferreira Gesteira, Vivien J. Coulson-Thomas, Yong Yuan, Jianhua Zhang, Helena B. Nader, Winston W.-Y. Kao

**Affiliations:** 1 Department of Ophthalmology, University of Cincinnati, Cincinnati, OH, 45267, USA; 2 Departamento de Bioquímica, Universidade Federal de São Paulo, São Paulo, São Paulo, Brazil

## Abstract

Lumican, a small leucine rich proteoglycan (SLRP), is a component of extracellular matrix which also functions as a matrikine regulating multiple cell activities. In the cornea, lumican maintains corneal transparency by regulating collagen fibrillogenesis, promoting corneal epithelial wound healing, regulating gene expression and maintaining corneal homeostasis. We have recently shown that a peptide designed from the 13 C-terminal amino acids of lumican (LumC13) binds to ALK5/TGFBR1 (type1 receptor of TGFβ) to promote wound healing. Herein we evaluate the mechanism by which this synthetic C-terminal amphiphilic peptide (LumC13), binds to ALK5. These studies clearly reveal that LumC13-ALK5 form a stable complex. In order to determine the minimal amino acids required for the formation of a stable lumican/ALK5 complex derivatives of LumC13 were designed and their binding to ALK5 investigated *in silico*. These LumC13 derivatives were tested both *in vitro* and *in vivo* to evaluate their ability to promote corneal epithelial cell migration and corneal wound healing, respectively. These validations add to the therapeutic value of LumC13 (Lumikine) and aid its clinical relevance of promoting the healing of corneal epithelium debridement. Moreover, our data validates the efficacy of our computational approach to design active peptides based on interactions of receptor and chemokine/ligand.

Proteoglycans (PGs) are complex polymers composed of a core protein to which one or more glycosaminoglycan (GAGs) chains are covalently attached. They play a central role in physiology and disease^1^,^2^,^3^,^4^,^5^,^6^: modulating cell metabolism, mediating cell-cell trafficking, providing support for connective tissue and regulating of a wide range of effectors at both systemic and cellular levels^7^. Small leucine rich proteoglycans (SLRPs) are a family of proteoglycans with small core proteins (~40 Kd) that contain hyper variable glycosylation sites with multivalent binding abilities^8^. To date, the SLRPs family encompasses 18 genes, which are classified into five distinct subfamilies based on homologies at both the genomic and protein levels^9^,^10^. Lumican, a class II SLRP member, is one of the major proteoglycans in the corneal stroma^11^,^12^. Members of the SLRP family, including Lumican, share similar structures and are composed of a 16-residue signal peptide, a negatively charged N-terminal domain, a ten-tandem leucine-rich repeat region (LRR), and a C-terminal domain containing 2 conserved cysteine residues. Each LRR has a conserved hallmark motif LXXLXLXXNXL (L: leucine which can be substituted by isoleucine, valine or other hydrophobic amino acid, while X indicates any other natural amino acid). This central LRR domain is responsible for forming the characteristic horseshoe-shaped solenoid structure with both convex and concave faces^9^. Both N- and C-terminus domains contain conserved cysteine residues, which maintain the protein core conformation and stability, and may also influence ligand binding^13^,^14^. A mutation at the C-terminal domain of Decorin has been linked to congenital stromal dystrophy of the cornea^15^,^16^. However, a disease directly related to a structural mutation of Lumican is yet to be confirmed. In the cornea, Lumican acts maintaining corneal transparency by regulating collagen fibrillogenesis and promoting corneal epithelial wound healing via a yet to be fully defined mechanism(s)^17^,^18^. Lumican may also serve as a matrikine, promoting corneal epithelial wound healing and maintaining corneal homeostasis by modulating gene expression in both physiological and pathological instances^12^,^19^,^20^. Recently, our group described the function of a synthetic LumC13-terminal peptide^6^, YEALRVANEVTLN (Lumikine), in binding ALK5 to promote wound healing^6^.

Herein, we exploited the C-terminal Lumican peptide surroundings, followed by further exploration of ALK5 as a potential target and further developed suitable peptidomimetics of LumC-peptides. Molecular docking and MD simulation studies were carried out to study the interaction of the LumC-peptides and ALK5 complex, which provides insights into vital binding interactions. These interaction studies not only revealed the relatively stable interaction of LumC peptide with ALK5 but also highlighted the significance of these interactions in activating ALK5. We were also able to establish the minimal amino acid sequence required for the formation of the lumican/ALK5 complex. Validations of the peptides functions in wound healing *in vivo* add to the therapeutic value of Lumikine in promoting healing of damaged corneal epithelium.

## Results

### Molecular Docking

**Figure 1:**
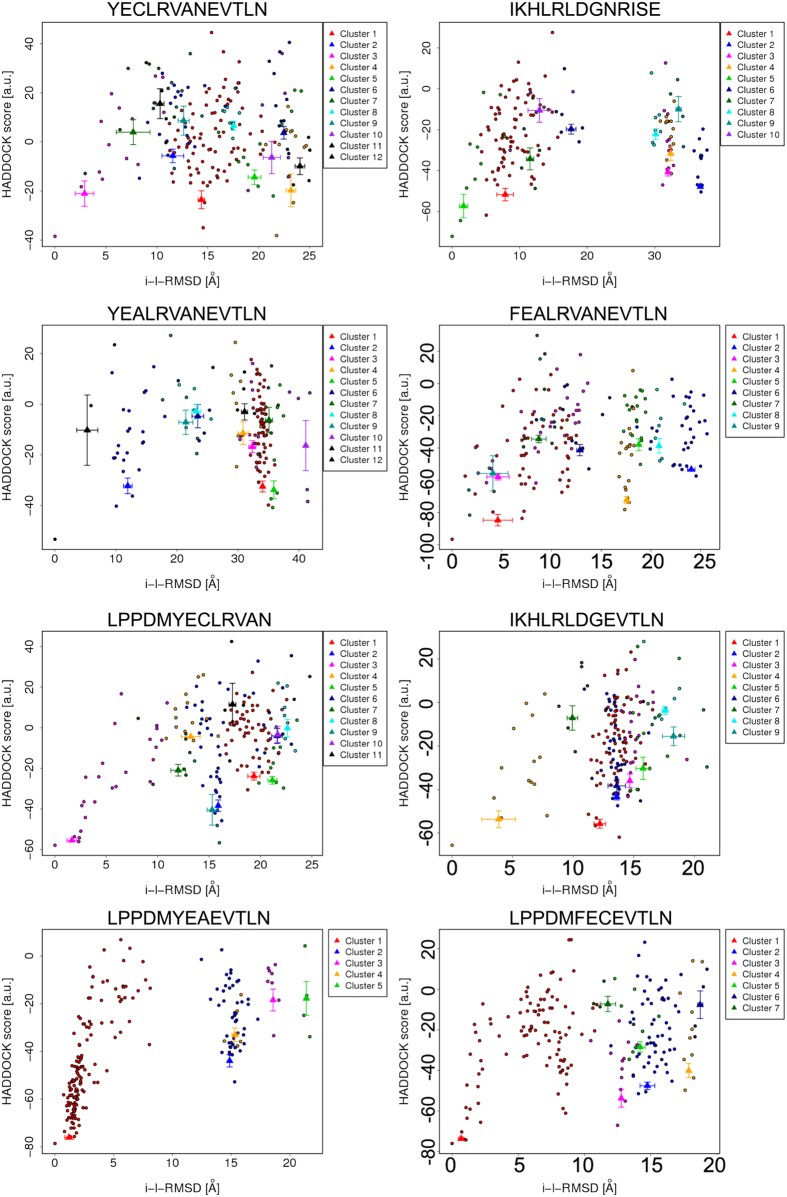
Scatter plots of global flexible docking results. Score values are represented versus RMSD over peptide backbone heavy atoms (rmsBB) with respect to the initial conformation. Score values are represented versus the ligand RMSD (l-RMSD). HADDOCK best scored poses (edge of tunnel) were subjected to MD simulations.

We used a modified HADDOCK benchmark for an efficient protein-peptide docking protocol, combining the conformational selection with induced fit^21^, where both receptor and ligand are independently minimized, as well as the starting protein–ligand interaction residues. In order to verify the specificity of the binding of the 13 C-terminal amino acids of lumican to ALK5 we designed 13 aa peptides based on the surrounding C-terminal region of lumican. Our data indicates the final 5 amino acids (EVTLN) were primarily involved in the LumC13-ALK5 interaction. To further verify this, we generated hybrid peptides, combining the EVTLN region with up-stream C-terminal regions, enabling us to ascertain these are the minimal amino acids necessary for LumC13-ALK5 interaction, the sequence of these peptides are summarized in Table 1. For the LumC peptides (Table 1), all 13 residues were specified as active residues, and for ALK5 a distance threshold of 15 Å around the GS domain was defined to select the residues that interact with the conformation obtained from previous work by Yamanaka, *et al*.^6^. The flexible docking performed with LumC13 returned 10 clusters, with 82 best poses in the best scoring cluster. This cluster (cluster 1, Fig. 1, Table 2 and Supplemental Table 1) had a HADDOCK score of −83.6 ± 7.3 kcal/mol. Overall, energies varied between −50 and −80 kcal/mol, indicating reasonable intermolecular affinities for all peptides docked.

As the HADDOCK score is determined for each structure after docking, a rank of the structures with the weighted sum of intermolecular electrostatic (Elec), van der Waals (vdw), desolvation (Dsolv) and AIR energies, and a buried surface area (BSA) was obtained, allowing for a comparison of values. While the LumC33_ΔC20_ score is −77.3 ± 11.4, Lumikine (LumC13_C-A_) and Lumikine_Y-F_ show a score of −83.8 ± 7.1 and −84.8 ± 7.0, respectively (Table 2). The less negative values of the HADDOCK score of the LumC33_ΔC20_ complexes indicate a lower affinity between the biological partners compared to the native complex, while the Lumikine_Y-F_ (LumC13_A,Y-F_) and Lumikine complexes have again a similar HADDOCK score as the LumC13 complex, illustrating again a good affinity between the biological partners. LumC18_ΔC5_ and the score of the hybrid peptides (LumC18_∆C5_, Hybryd 1/3, Hybryd 2/3 and Hybryd 2/3_Y-F_) amount to similar score values of −79.5 ± 1.7, −55.8 ± 4.0, −76.0 ± 1.6 and −82.5 ± 0.5, respectively. The BSA is used to quantify the protein surfaces, which are not exposed to water. The LumC13 complex shows a BSA value of 1490.8 ± 109.1, while the BSA values of the LumC33_ΔC20_, Lumikine, Lumikine_Y-F_ are 1375.6 ± 140.2, 1384.9 ± 79.8 and 1574.4 ± 138.3, respectively, and the LumC18_ΔC5_ and the score of the hybrid peptides exhibit a BSA score of 1450.3 ± 71.0, 1266.2 ± 138.5, 1441.9 ± 35.8 and 1284.2 ± 24.2, respectively (see Table 2 and Table S1). A higher BSA value enables a close proximity between the biomolecules. The desolvation energy, the restraints violation energy and the BSA have a good correlation with the docking score of the complex during docking. From Table 2, it is thus again clear that both LumC_13C-A_ and Hybrid 2/3 peptides would present higher affinity to ALK5, whereas hybrid 1/3 shows the lowest interaction values to ALK5.

### Molecular Dynamics

Molecular dynamics (MD) calculations have been previously used in understanding and characterizing the structural, dynamical, and functional properties of biomolecular systems. Our group has recently developed its application for the rational design of lumican peptides capable of binding ALK5^6^.

**Figure 2:**
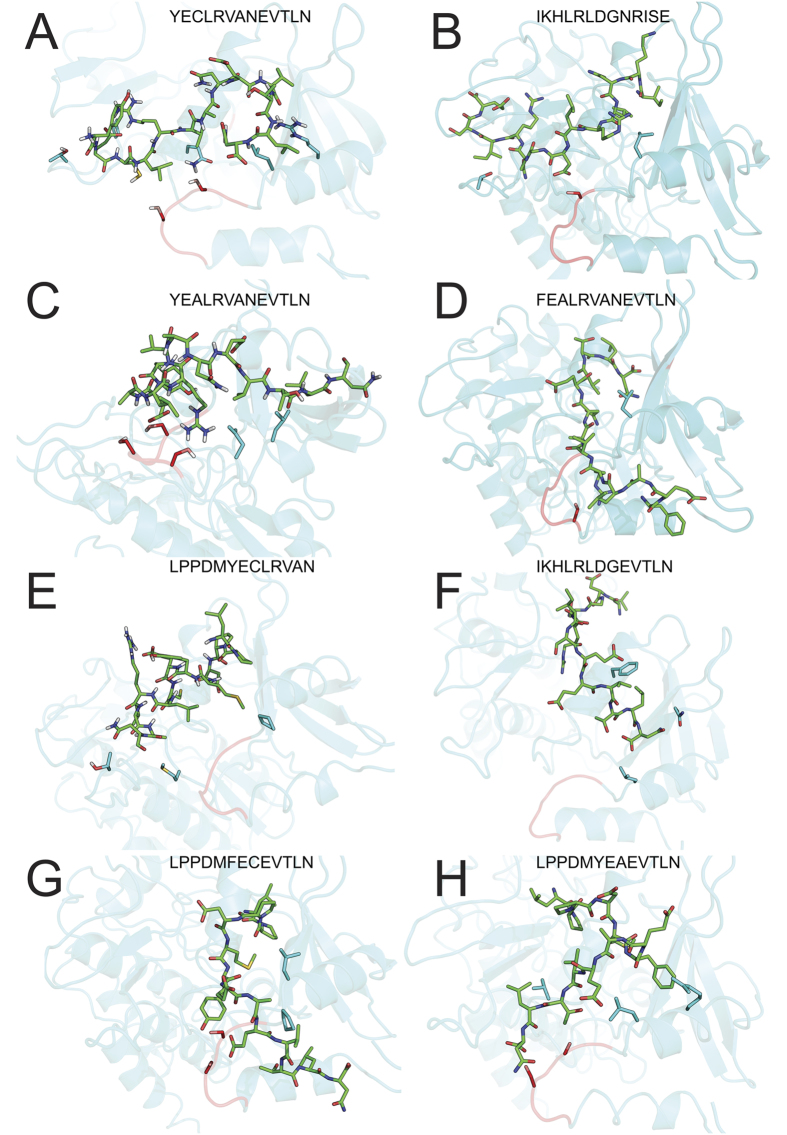
C-terminal Lumican peptides bind to the GS-region of ALK5. Peptides average structures after minimization bound to ALK5 as depicted in Table 1. ALK5 shown as cyan cartoon, peptides shown as green sticks and GS-region depicted in red. Serine forming hydrogen bonds with peptides shown as red sticks. (**A**) LumC13; (**B**) LumC33_Δ20_; (**C**) Lumikine; (**D**) Lumikine_Y-F_; (**E**) LumC18_ΔC5_; (**F**) Hybrid1/3; (**G**) Hybrid1/3; (**H**) Hybrid2/3; (**I**) Hybrid2/3_Y-F_.

Eight sets of 100-ns simulations were performed for each of the peptide-ALK5 systems described above. All simulations were performed under constant temperature and pressure conditions. Visual inspection of 100 ns MD trajectories clearly revealed that LumC33_ΔC20_ monomer essentially cluster within mixed conformations, specifically around the C-terminus, leading to a large backbone root-mean-square deviation (rmsd) of 2.3 Å. We next clustered the similar conformations of hybrid peptides in comparison with LumC18_ΔC5_ using the cluster plugin in VMD^22^ with the criteria of a Cα rmsd cutoff of 1.0 Ǻ. Figure 2 shows each peptide most representative conformations populated. Each cluster represents a comparable structural population of ∼3.3%, representing a sum of ∼16.3% of all conformations. It can be seen that most representative hybrid 2/3 peptide conformations displayed little transit between conformations, with 89.4% of the second conformation of the same cluster grouped (Fig. 2G). It is reasonable to infer from this structural property that this peptide occurs in an oriented mode to ALK5, as opposed to the hybrid 1/3 (Fig. 2F), suggesting that this peptide forms a complex adopting multiple conformations when bound to ALK5. Moreover, the GS-domain appears to be stabilized by contacts among Glu2, Glu9 and Asn8 from LumC13_C-A_ and Lumikine_Y-F_, while the mutation of a Tyr1 to phenylalanine in peptide Lumikine_Y-F_ does not affect binding significantly. Those residues bind specifically to the GS-domain region (Fig. 2C,D and Supplementary Fig. 1).

The contact probability per amino acid type analysis reveals that both positively and negatively charged residues (Arg, Lys, Glu) of the peptides are involved in ALK5 binding, while significant participation of the polar (Ser, Thr), and hydrophobic (Leu, Pro, Val) residues is also observed. These results imply that the LumC33_ΔC20_ contact surface is more homogeneous in terms of the peptide amino acid composition, as no particular amino acid type shows a strong binding preference for LumC33_ΔC20_.

The higher contact probability of the mainly hydrophilic LumC13_C-A_ is consistent with their frequent interaction with the GS domains of ALK5. Given the more heterogeneous nature of the hybrid surface, it is likely easier for the hybrid N-terminus tyrosine to find a complementary binding pocket that can satisfy all or majority of the possible charge-charge interactions. Thus, the C-terminus (with a smaller local concentration of charged residues) appears to remain in contact with GS domain more frequently compared to the N-terminus.

Further analysis reveals that this difference is caused by the different flexibility of hybrid 2/3 conformations sampling, reflecting its entropy. As shown in Fig. 2, while the methionine serves as the main anchoring residue during simulations, the remaining residues have maintained contact profiles.

### Molecular Dynamics simulations

LumC13, a peptide that binds to ALK5 and peptides derivate from rational design were evaluated regarding their binding/interacting potency^6^,^23^,^24^, to explore their potential binding modes with ALK5 and find out the causes of the difference from a theoretical perspective via molecular docking, molecular simulation, and MM-PBSA calculation. We determined the optimal binding modes of the LumC peptides with ALK5 by 100 ns molecular dynamics simulations based on the docking results to explore the binding mechanism. Then, we ran triplicates of 100 ns MD for each complex, and conformations were analyzed for analogous differences of key residues around their apparent binding sites. In the following sections, the results from one of the triplicates were discussed. First, the RMSD of backbone C_α_ atoms were analyzed to determine if these systems reached equilibrium.

In Fig. 2A (Ligplot in Supplemental Fig. S1), the detailed binding modes of LumC13 peptide reveals that the carbonyl oxygen atom and carboxyl group of the Glu9 of LumC13 establish hydrogen bonds with the Gly188 of ALK5 main chain. The Arg5 of LumC13 is stabilized by its main chain with Gly190, Gly325 and Met253 via hydrogen bonds. The side chain of Tyr1 of LumC13/Lumikine is stabilized with residues ALK5 Glu247 and Gln250 by hydrogen bonding interaction. Finally, Asn8 of LumC13/Lumikine is anchored by hydrogen bond with ALK5 Ser187.

The probability of occurrence of hydrogen bonds was calculated by using the H-bonds plugin v1.2 in vmd^22^ and the corresponding results of H-bonds between peptides and ALK5 are shown in Table 3. As the occurrence probability of the aforementioned hydrogen bonds are reasonably high (three higher than 90%, two higher than 65%), this suggests these H-bonds exist stably. The existence of these H-bonds might lead to strong electrostatic interactions between receptors and donors, which will be demonstrated by results of MM-PBSA calculation.

Figure 2B shows that in the LumC33_ΔC20_-ALK5 complex system, one pair of hydrogen bonds exist between the side chain of Lys2 and the carboxyl of Glu247 and Glu250. The carbonyl oxygen atom of Glu247 also interacts with Ile1 through hydrogen bond. The side chains of Arg5 and Leu6 tend to form hydrogen bond with Ser191. In addition, the hydroxyl of Thr251 establishes a hydrogen bond with the N-gama nitrogen of His3 of LumC13.

**Figure 3:**
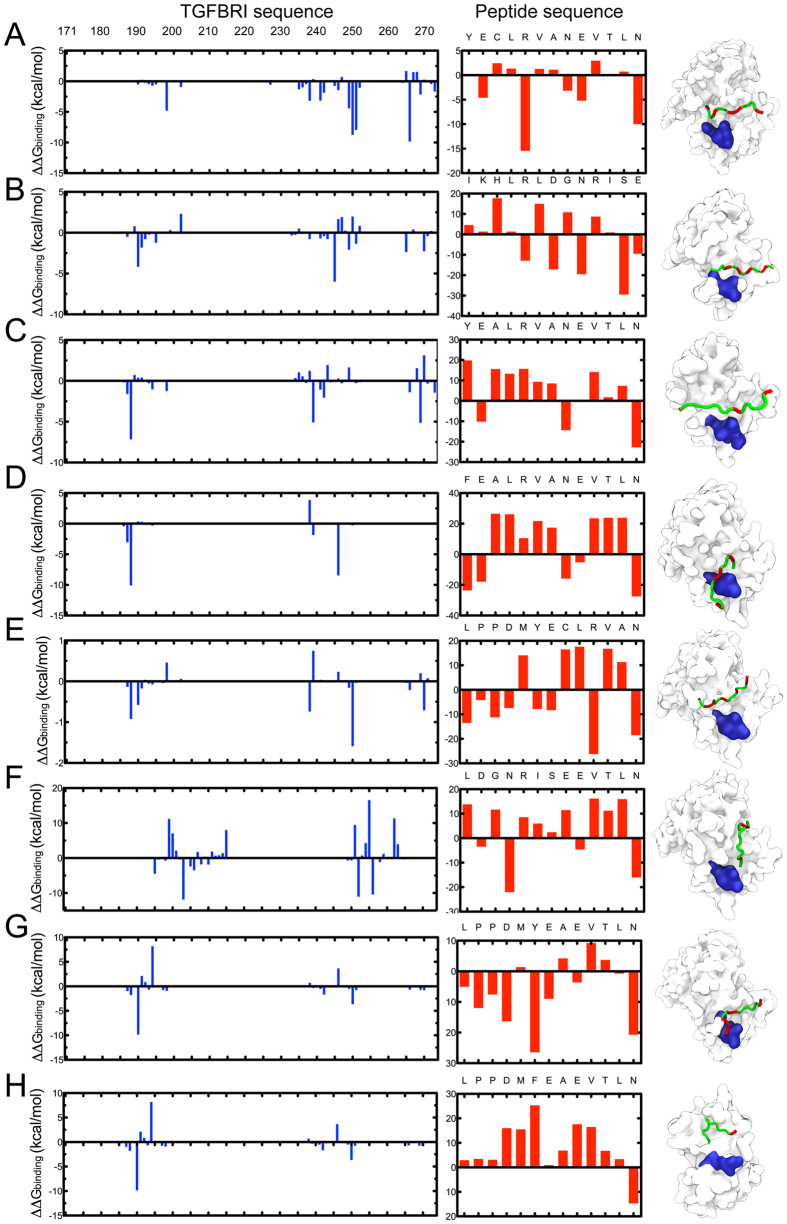
Per-residue energy contributions to the formation of lumican-ALK5 complexes. Free energy of bound lumikine and lumican peptides to TGFBRI/ALK5. ALK5 residues are shown as blue lines and peptide shown as red bars. Error bars were not included since standard errors were always.

In order to further validate the binding modes, we carried out molecular dynamics simulations to gain detailed information about the key residues of the active site in each complex, the electrostatic, van der Waals, solvation and total contribution of the residues to the binding free energy of Lumikine–ALK5 and LumC-peptides-ALK5 interaction were calculated with the MM-PBSA method. The contributions from the residues around the GS-domain are shown in Fig. 3 and Table 3.

### Binding energy estimation by MMPB(GB)SA

Several techniques exist to predict binding free energies based on MD simulations, including free energy perturbation, thermodynamic integration, molecular mechanics Poisson–Boltzmann surface area (MMPBSA), and molecular mechanics generalized Born surface area (MMGBSA) techniques^25^. Although recent studies reported encouraging results for the Free-energy potential method in the prediction of relative ligand binding potency, its application in lead optimization is still at the very early stage for therapeutic drug discovery^26^.

MMPB(GB)SA approaches have become standard for estimation binding free energies since they offer a good balance between speed and accuracy. MMPB(GB)SA combines force field based molecular mechanics (MM) with a continuum model, based on the Poisson–Boltzmann (PB) or generalized Born (GB) equation to handle electrostatic interactions, and a nonpolar solvation energy correlated with solvent accessible surface area (SA). This method can be applied to any aqueous binding process: it is not dependent on assumptions of small (perturbative) differences between two similar systems. The configurational entropy due to ligand–protein binding can be calculated by a normal mode (NMODE) method. The MMPB(GB)SA methodology has been applied in various small molecule–protein systems, including drugs binding to their receptors^26^,^27^,^28^. The approach we described here is most closely associated with this method.

The contribution of selected residues surrounding the GS domain active site can be clearly seen from Fig. 3. For LumC13, Arg5 and Asn13 have significant total energy contributions. Although there are many factors for the formation of hydrogen bonds, electrostatic interactions play a major role. As described above, the relationship between the hydrogen bond and electrostatic term are discussed qualitatively. In fact, several hydrogen bonds are easily formed because of strong electrostatic interactions. From the ALK5 GS domain region, Glu247, Ser187, Gly192, Thr251 and Ser191 have prominent electrostatic contributions evidenced by lower values of ΔE_ele_ (−4.97, −7.75, −26.15, and −47.13 kcal mol^−1^, respectively), and are correlated to the formation of several hydrogen bonds with LumC13 qualitatively, as shown in Fig. 2 and Supplemental Fig. S1.

The strongest electrostatic contribution comes from the GS domains and the helix 8 located on its opposite site, which promotes one strong H-bond in complex. The lowest averaged value of ΔE_ele_ (−7.13 kcal mol^−1^) implies that Ser187 might make a special contribution for active site of GS-domain. Strong electrostatic interactions result in the formation of hydrogen bonds between ALK5 and Asp7 and Glu2 in LumC13, LumC13_C-A_ and LumC13_AY-F_.

LumC13 Asp9 has the strongest electrostatic contribution, which promotes one strong H-bond in complex. The lowest value of ΔE_ele_ (−47.13 kcal mol^−1^) implies that this residue might make a special contribution for peptide binding. Peptides outside EVTLN domain region reduced the binding by 10,000- fold (value of k_cat_/K_m_ is 1.0 ± 0.004 and <0.0001 s^−1^ μM^−1^ in LumC13 and LumC33_ΔC20,_ respectively. In addition, Val10 and Leu12 of LumC13/Lumikine are close to ALK5 Gly182, and two electrostatic interactions exist, which leads to two H-bonds. Ser180, with a ΔE_ele_ of −7.75 kcal mol^−1^, has two electrostatic interactions with the ligand because of the close proximity between Gly183 and Val3, which promote two H-bonds, as shown in Fig. 3.

The van der Waals contribution of some residues in the region surrounding the GS-domain, particularly Gln250 and Thr251, play the main role in the total protein-peptide contributions (Fig. 3A–F). Of these residues, Gln250 has the strongest van der Waals contribution of −3.76 kcal mol^−1^. In addition, the free energy calculation results show that ALK5 Thr251 van der Waals contributions are also significant to the system as a whole. Throughout the energetic decomposition and probability of occurrence of hydrogen bonds, we determined that the most fundamental binding residues in the ALK5 GS domain were Asp183, Gly188, Ser189, Gly190, Ser191, and Ser187. The major contributions of Gly-Ser region originate from electrostatic interactions and those of Asp183 and Gln247 were from a van der Waals’ interactions. Figure 3 shows residues at binding sites with appreciable electrostatic contributions, with all ΔE_ele_ of <−20 kcal mol^−1^. Strong electrostatic interactions result in the formation of hydrogen bonds between ALK5 and peptides containing the EVTLN region. It is noted that Y6 from peptide Hybrid 2/3 also makes a positive electrostatic contribution to the system. However, with Hybrid 2/3_Y-F_ there is no H-bond formation between Phe9 and the residues around the GS domain.

Although there is no direct H-bond forming with Hybrid 2/3_Y-F_, Pro2 and Pro3 electrostatic contributions secure peptide binding to the GS-region. Strong electrostatic interactions might result in the formation of hydrogen bonds between these residues and the GS-domain as depicted in Fig. 3. ALK5 Ser189 has the highest van der Waals contribution at −12.53 kcal mol^−1^. Throughout the whole energetic decomposition and probability of occurrence of hydrogen bonds, we determined that the most vital binding residues at the GS-domain were the ones before mentioned.

When the decomposition of the binding energy of the residues in both domains were compared, we found that the similar van der Waals’ interactions are found in peptides containing the EVTLN region. Nonetheless, the substitution of Y9 to F mutation in Hybrid2/3_Y-F_ has an evident impact in electrostatic forces for the GS-domain binding; however, the magnitude of electrostatic term in the C-terminal domain was larger. The strength of the binding ability between each peptide and GS-domain cannot be determined clearly so far; thus, it is necessary to call for further studies and discussion about total binding energy of these complex systems. As mentioned above, the total binding energy of ALK5–LumC peptides complex systems are an important standard measure of binding affinity between each domain and ALK5. Total binding free energies for the two complex systems were calculated by MM-PBSA. The MMPBSA results are listed in Fig. 3. The ΔG_total_ can be divided into polar and nonpolar energies. The free energies of ALK5 binding to the peptides were primarily derived from the polar energies (equal to ΔG_ele,sol_ + ΔE_ele_), which were −218.0 kcal mol^−1^ in LumC-ALK5 complexes and −446.3 kcal mol^−1^ in Hybrid 2/3–ALK5 complex (Fig. 3). However, for both complexes, the nonpolar energy (equal to ΔG_nonpolar,sol_ + ΔE_vdw_) shows negative contributions at 134.9 and 378.3 kcal mol^−1^ for LumC13 and Hybrid 2/3_Y-F_, respectively, which largely cut down the total contribution. Thus, the values of ΔG_total_ for LumC13 and Hybrid 2/3_Y-F_ are −83.1 and −68.0 kcal mol^−1^, respectively, which indicates that ALK5 has a stronger binding interaction to this hybrid than the LumC13/Lumikine peptide. We qualitatively studied the difference in terms of binding energy between the peptide and ALK5 domains; which indicated that the LumC13 has a stronger ability than the N-terminal peptides to bind to ALK5, which is in good agreement with our conclusion. In addition, the relative affinities of the C-terminal active sites of Lumican were very similar; thus, the difference in total binding energy between each domain and ALK5 might just be consistent with that C-terminal region.

In short, computational methods identified peptide Hybrid 2/3 as capable of binding ALK5 domain with the lowest interactions energies, and that binding depends on the EVTLN domain. The computational studies predicted that although LumC peptide derivatives bind to ALK5 binding and pose as a potential new target for promoting wound healing. In order to further improve the peptides affinity, we subsequently attempted to modify and optimize the primary sequence of the peptide. The designed peptides were assayed *in vivo* to determine capability to promote wound healing using a debridement wound healing model. As our computational studies predicted, peptides Lum13/Lum_C-A_ and Hybrid 2/3 exhibit an increased ability to promote wound healing. Hybrid 2/3 possesses the highest affinity in all designed peptides (K_d_ = 28.7 ± 3.9 lM), and thus can be considered as a promising lead to develop peptide therapeutics targeting wound healing.

### Peptide Delivery via Eye Drop to Mouse Cornea after Epithelial Debridement

To confirm peptide action promoting corneal epithelium wound healing *in vivo*, mice were subjected to a 2 mm with corneal epithelium debridement. The injured corneas were treated with 10 μl eye drops lumican peptides (0.3 μM) every 15 min for 5 h as previously described^6^. Migration distance was measured between the original wound edge and the wound front. In Lumikine (LumC13_C-A_) treated corneas, the epithelium sheet moved ∼144 μm. The control peptide, LumC_30_Δ_C20_, did not promote epithelium wound healing, whereas peptide hybrid 2/3 promotes wound migration of about 107 μm. The average epithelium migration (μm) and standard deviation were calculated from n corneas of each of two separate experiments.

**Figure 4:**
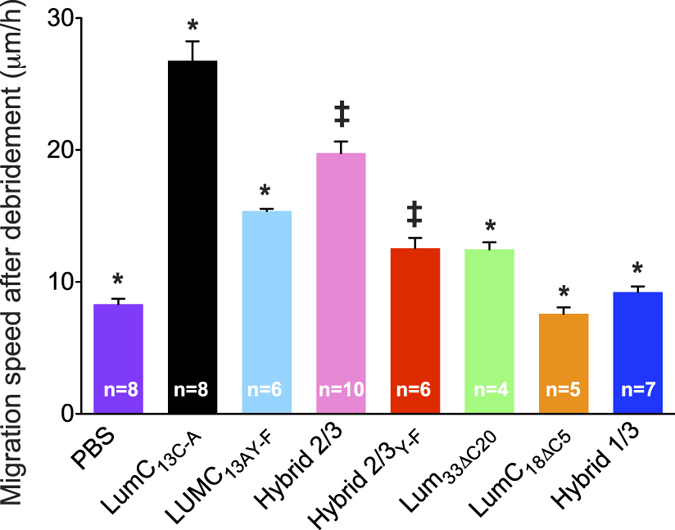
*In vivo* wound healing activity of Lumican peptides. Corneal epithelium debridement wound healing *in vivo*. The average epithelium migration per hour (μm/h) and standard deviation were calculated from n corneas of each of two separate experiments. *<0.005; ^‡^Hybrid 2/3 is 0.092 that is p < 0.05; LumC13A Y-F is 0.122 that is p < 0.05. Statistical analysis was performed using ANOVA.

Lum null (*Lum*^−/−^) mice were also used to examine the efficacy of LumC peptides in promoting epithelium sheet migration following debridement wound (Supplemental Fig. S2). Figure 4 shows that the administration of LumC13_C-A_ promoted wound healing of corneal epithelium debridement of *Lum*^−/−^ mice to the same extent as injured wild type mice as previously shown^6^. Treatment with Hybrid 2/3 also increased the epithelium migration (~110 μm) while the substitution of Y to F abolished the epithelial migration promoting activity. Interestingly, administration of LumC33_Δ20_, LumC18_ΔC5,_ and Hybrid1/3 had little effect promoting wound healing, suggesting that the C-terminal domain of EVTLN is essential for promoting epithelium migration of injured cornea. LumC13 had moderately increased activity in promoting wound healing when compared to LumC13_C-A_. Mass spectrometry revealed the higher pharmaceutical activity of LumC13_C-A_ compared to LumC13 was due to the dimerization of the LumC13 peptides in solution via disulfide bond formation of cysteine residue, which reduces the activity of dimerized LumC13. The summarized data from different treatments shows each treatment including at least five or more eyes from two separate experiments. These results showed that both the LumC13_C-A_ and Hybrid 2/3 peptides significantly promoted epithelium wound healing.

## Discussion

This study depicts the different binding energies of different 13 amino acid LumC peptides to ALK5 by combining molecular dynamics results and docking conformational changes. Our computational data was then confirmed *in vivo* using a corneal epithelial wound healing model. In all models, several pairs of hydrogen bonds are found in the region around the ALK5 GS-region, such as the hydrogen bond between the N of residue Gly188 and hydroxyl side-chain oxygen of LumC13 Glu9 and the hydroxyl of Thr251, as well as the side chain oxygen of Gln250. Analyses of contribution of the binding free energy and its decomposition into per-residue indicate that the conformational changes of the region around the GS-domain region is a receptor for binding of the Lumikine.

Injured mouse corneal epithelial cells ectopically and transiently expresses lumican during the acute phase of wound healing^18^,^29^,^30^,^31^,^32^,^33^. Thereafter, the degradation of Lumican by MMPs within the extracellular space of the corneal epithelium^34^,^35^, such as MT1-MMP, leads to the distribution of Lumican products throughout the wounded area, which could have potential pharmacological relevance. Therefore, we explored the potential of Lumican peptides to bind to a previously established Lumican surface receptor, ALK5/TGFbRI, which has been shown to strongly enhance skin and cornea wound healing^6^,^36^.

We have previously shown that Lumican promotes wound healing via its C-terminal domain due to its interaction with TGFβRI (ALK5), preventing further canonical TGFβ signaling via Smad cascade following polymerization of TGFBR2 and ALK5/TGFBR1^6^. In this present study, we have accessed the binding of rationally designed LumC peptides with a related scoring approach, MM-GBSA, after docking and molecular dynamics simulations. In this method, docking scores are used to define initial binding sites, and more expensive and rigorous methods such as molecular dynamics (MD) simulation and binding free energy (BFE) calculations are employed to study these interactions^37^,^38^,^39^. These computational methods are considered accurate, as factors such as flexibility of the complex and solvation effects are taken into account. Whereas several studies have shown the successful application of binding free energy approaches in predicting binding modes for diverse molecules at binding pockets of target proteins^40^,^41^,^42^. However, a comprehensive streamline methodology of rational design, *in silico* evaluation and validation have not yet been widely applied to predicting the binding mode of protein–peptide complexes in their potential use as inhibitors.

Here, we evaluated the binding free energy after decomposition into per-atom contributions, which could be summed over atom groups to obtain different energy contributions arising from residues backbone and side-chain by using the MMGBSA method, which is particularly useful to explore the variability of these peptides. After applying *in silico* methodology, peptides were successfully tested experimentally for their propensity to promote *in vivo* wound healing after binding TGFBRI. The most potent of these peptides exhibited activity in an *in vivo* model of corneal epithelium debridement. With the *in vitro* evidence for modeling experiments, our analysis suggests that lumikine binding is targeted to the GS-domain of ALK5. Conclusions based on these simulations should take into account the effect of peptide initial conformations; although multiple copies of trajectories on the 100-ns timescale (enhanced sampling) may still affect the simulation results. The exploration of the contribution of binding free energy and its decomposition into per-residue analysis indicate that the conformational changes of the region around the GS-domain play an important role in understanding of the binding affinity to the LumC peptides.

Our data also demonstrates great pharmacological applications of using LumC13 derivatives for promoting the rate of wound healing. This could be of particular interest in conditions where patients display delayed wound healing, such as diabetes, which culminates in an increased risk of inflammation and scarring. The significance of synthesizing lumican peptides that mediate TGFbRI signaling also goes beyond promoting skin and corneal wound healing. We have previously demonstrated that lumican inhibits prostate cancer cell migration, and thus is mediated through the TGFβ signaling pathway^1^,^3^. Our group recently reviewed the role of SLRPs, including lumican, on pathogenesis^5^. Thus, Lum peptides could also be targeted for other malignancies such as limiting the spread of certain types of cancer cells.

Our approach represents a fast and straightforward approach that can be applied as a general method to determine binding modes of protein−peptide complexes and also to design TGBβRI inhibitors.

## Conclusion

An *in silico* approach combining molecular docking and molecular dynamics was successfully used to predict minimal lumican amino acid sequence necessary for forming a stable complex with ALK5/TGFbRI. *In vitro* and *in vivo* wound healing assays demonstrated that LumC13, markedly the EVTL motif, is essential to promote epithelial cell migration to the same extent as lumican, thereby confirming the *in silico* data.

## Experimental Section

### *In vivo* healing of epithelium debridement

Use of experimental mice is in compliance to the ARVO statement for the use of animals in vision research and all procedures are approved by IACUC of the University of Cincinnati. *Lum*^−/−^ mice in C57BL/6 genetic background aged 7–8 weeks were used in this study. Mice were anesthetized with intraperitoneal injections of a combination of ketamine (10 mg/kg; Henry Shein) and xylazine (1 mg/kg; Henry Shein) prior to the surgery, and a drop of proparacaine hydrochloride 0.5% (Alcon) was applied to the cornea to deliver local anesthesia before injury. Briefly, a 2 mm diameter trephine was used to demarcate the central epithelial region of the eye and the epithelium within the demarcated region was mechanically removed using an Algerbrush II (Alger Equipment Co., Inc., Lago Vista, TX) while viewing the cornea under a stereomicroscope (Stemi SC II stereomicroscope with epi-fluorescence attachment, Carl ZEISS). Eight mice per group were treated and the experiments were repeated at least two times. Each mouse was topically treated with one drop peptide solution (10 μl) at concentration of 0.3 μM or 10 μL PBS every 15 min for 5 h after wounding. After 5 h, mice were sacrificed. The eyes were excised and fixed in 4% paraformaldehyde in PB (0.1 M phosphate buffer, pH 7.4. whole mount corneas were stained with phalloidin and DAPI and image with a Zeiss Apo Tome microscope (Observer Z1). The migration of epithelium sheet was determined as previously described^5^,^6^. In brief, migrated corneal epithelium was measured on whole mount samples in 5 different locations equally distributed around the entire circumference of the wound. The number of animals in each experimental group is on the bars in the bar chart. The statistical analyses as well as symbols are shown in the graph legend. PBS treated Lum^−/−^ mice were used as negative controls, in which the epithelium sheet migrated less than 50 μm in 5 h (Supplemental Fig. 2 for representative plots). Four Z-staged images were collected from each corneas, the epithelium migration was determined by the distance of migration of epithelium front to the initial injured line as previously described.

### Peptide–protein flexible dockings

The peptide sequence used in the ALK5 docking studies was derived from a part of Lum C-terminal region consisting of residues from amino acids 321–338, as it was previously used in the activity measurements conducted by our group^6^,^43^. The ALK5 complex models were generated using a two-phase docking protocol using HADDOCK web server http://haddock.chem.uu.nl/44. In the first phase, the LumC peptides were docked onto the structure of ALK5 with a three-step protocol for performing the docking calculations. First, one thousand structures were generated during rigid body docking stage, of which the best 100 structures based on their intermolecular energy were submitted to the second stage of semi flexible refinement. All 100 structures were then refined in the third stage using an explicit solvent (in water) and were clustered based on the root mean square deviation (RMSD). HADDOCK scoring was performed according to the weighted sum (HADDOCK score) of different energy terms, which include van der Waals energy, electrostatic energy, distance restraints energy, inter-vector projection angle restraints energy, diffusion anisotropy energy, dihedral angle restraints energy, symmetry restraints energy, binding energy, desolvation energy and buried surface area. Along with the HADDOCK score, we calculated the van der Waals energy, electrostatic energy, restraints violation energy, desolvation energy and buried surface area.

The best pose clustered in the best binding region returned by the rotational blind docking was used as starting conformation. Accordingly, flexible docking protocols Rosetta FlexPepDock Web Server^45^ and Cluspro^46^ were used to study and compare the allosteric interactions retrieved among themselves. For the eight ALK5/peptide complexes, the top ten poses with the highest scores were found to be very similar, and the one with the highest score was chosen for refinement with MD. Hybrid peptides were rationally designed by including other regions of the C-terminal domain of Lumican that bind to ALK5^6^ and modeled using PEP-FOLD^47^.

Each complex structure solvated with 17,216 water molecules and 0.1 M KCl. After pressure coupling, individual protein-peptides complexes were gradually relaxed in 50-ns MD simulations, where convergence was monitored using the RMSD of the backbone atoms. MD simulations were performed using the NAMD program^48^ with the CHARMM27 force field^49^. An NpT ensemble was used with the temperature and pressure maintained at 300 K and 1 atm, respectively, via Langevin coupling with damping coefficients of 5 ps^−1^ and 10 ps^−1^. Periodic boundary conditions were employed together with particle-mesh Ewald algorithm to compute the long-range electrostatic interactions. Lennard-Jones (LJ) interactions were switched off within a distance of 10–12 Å, and the list of non-bonded interactions was truncated at 13.5 Å. A time step of 2 fs was used, and the trajectory data were written at 1 ps intervals. Intermolecular contacts (hydrogen bonds and non-bonded contacts) were analyzed with the LIGPLOT software^50^. The default settings were used (3.9 Å heavy atoms distance cut-off for non-bonded contacts; 2.7 and 3.5 Å proton–acceptor and donor–acceptor distance cut-offs, respectively, with minimum 90° angles (D–H–A, H–A–AA, D–A–AA) for hydrogen bonds)^51^.

MM/PBSA binding free energy calculations: To determine the most stable complexes predicted by the molecular docking, the binding free energy (ΔG_bind_) for each complex was estimated by using the MM/PBSA approach previously based on the snapshots extracted from the single trajectory of the complex (single trajectory method)^25^,^52^, producing precise and reproducible free energy estimates for peptide-protein simulations^28^,^53^,^54^. Accordingly, the binding free energy of the peptides to ALK5 in solution was defined by the following equation^55^.


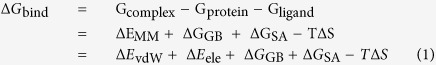


where ΔE_MM_ is the interaction energy between protein and ligand in the gas-phase, including the parts: the van der Waals energies (∆E_vdw_) and the electrostatic (∆E_ele_); ∆G_GB_ and ∆G_SA_ are the relative polar and nonpolar contributions to desolvation free energy, respectively, and [−T∆S] represents the conformational entropic contribution at temperature T. In this work, ΔG_polar_ and ΔG_nonpolar_ were calculated with APBS^56^. Finally, the binding free energy of each system was calculated based on 300 snapshots from the last 30 ns MD simulation trajectories of each complex by using the mm_pbsa program in Gmxpbsa^57^.

### Free energy decomposition analysis

To properly evaluate individual residue influence in the interaction between ALK5 and LumC peptides, a free energy decomposition process was applied to the mm_pbsa analysis. During the decomposition process, the polar contribution of desolvation free energy (∆G_GB_) was calculated using the generalized Born (GB) approximation model developed by Onufriev *et al*.^45^, and the nonpolar solvation contribution (∆G_SA_) part was computed based on the SASA determined with the ICOSA algorithm^58^. Energy components were from snapshots as described above, resulting in residue-allocated peptide free energy contributions from the association with its receptor.

## Additional Information

**How to cite this article**: Gesteira, T. F. *et al*. Lumican Peptides: Rational Design Targeting ALK5/TGFBRI. *Sci. Rep.* **7**, 42057; doi: 10.1038/srep42057 (2017).

**Publisher's note:** Springer Nature remains neutral with regard to jurisdictional claims in published maps and institutional affiliations.

## Supplementary Material

Supplementary Information

## Figures and Tables

**Table 1:** Sequence of Lumican Peptides Designed/Analyzed.

Lumican Peptide	Amino Acid Sequence
LumC13	**YECLRVANEVTLN**
LumC13_C-A_ (Lumikine)	**YEALRVANEVTLN**
LumC13_AY-F_	**FEALRVANEVTLN**
LumC18_ΔC5_	**LPPDMYECLRVAN**
LumC33_ΔC20_	**IKHLRLDGNRISE**
Hybrid 1/3	**IKHLRLDGEVTLN**
Hybrid 2/3	**LPPDMYECEVTLN**
Hybrid 2/3_Y-F_	**LPPDMFECEVTLN**

**Table 2:** Statistical analysis of the protein-peptide docking result obtained by HADDOCK.

Peptide	HADDOCK score	Van der waal energy	Electrostatic energy	Desolvation energy	Restraints violation energy	Buried surface area
		(Kcal mol^−1^)	(Kcal mol^−1^)	(Kcal mol^−1^)	(Kcal mol^−1^)	(Å_2_)
LumC13	−83.6 ± 7.3	−40.9 ± 6.3	−436.1 ± 24.2	42.4 ± 3.6	21.8 ± 15.74	1290.8 ± 109.1
LumC33_ΔC20_	−77.3 ± 11.4	−45.5 ± 8.0	−231.0 ± 23.3	31.1 ± 2.8	32.9 ± 9.99	1375.6 ± 140.2
LumC13_C-A_	−83.8 ± 7.1	−43.4 ± 3.5	−153.0 ± 29.2	36.0 ± 7.2	42.1 ± 27.24	1384.9 ± 79.8
LumC13_A,Y-F_	−84.8 ± 7.0	−49.8 ± 7.1	−313.1 ± 40.4	23.8 ± 16.6	37.4 ± 28.55	1574.4 ± 138.3
LumC18_ΔC5_	−79.5 ± 1.7	−48.2 ± 7.2	−453.9 ± 27.8	42.4 ± 6.1	10.4 ± 5.86	1450.3 ± 71.0
Hybrid 1/3	−55.8 ± 4.0	−41.7 ± 9.0	−307.5 ± 57.5	46.6 ± 10.6	7.9 ± 3.80	1266.2 ± 138.5
Hybrid 2/3	−76.0 ± 1.6	−55.2 ± 2.8	−384.7 ± 11.5	15.5 ± 3.3	6.5 ± 2.02	1441.9 ± 35.8
Hybrid 2/3_Y-F_	−82.5 ± 0.5	−44.1 ± 2.5	−270.4 ± 11.8	24.8 ± 3.9	9.1 ± 2.89	1284.2 ± 24.2

**Table 3:** Hydrogen Bond occupancies for the lumikine peptides-ALK5 complex.

Peptide	Acceptor	Donor	Presence (%)
LumC13	Gln N-H	Tyr OH	94.37%
LumC30_ΔC20_	Gly247 COO-	Arg N-H	61.29%
LumC_13C-A_	ValC-O	Gln N-H	89.35%
LumC_13AY-F_	ValC-O	Gln N-H	66.94%
LumC_18ΔC5_	Asp COO-	Lys N-H	52.19%
Hybrid 1/3	ValC-O	Lys N-H	63.44%
Hybrid 2/3	Asp COO-	Lys N-H	71.84%
Hybrid 2/3_Y-F_	Asp COO-	Lys N-H	87.15%
