# Supplementary material for: Lumican Peptides: Rational Design Targeting ALK5/TGFBRI
Source: Sci Rep. 2017 Feb 9;7:42057. doi: 10.1038/srep42057 (PMC5299588; doi:10.1038/srep42057)
Supplement: Supplementary Information [file srep42057-s1.pdf]

Lumican Peptides: Rational Design Targeting ALK5/TGFBRI

Tarsis Ferreira Gesteira[1]*, Vivien Jane Coulson-Thomas[1]*, Yong Yuan[1], Jianhua Zhang[1], Helena B. Nader[2] and Winston W-Y Kao[1]

[1]Department of Ophthalmology, University of Cincinnati, Cincinnati, OH, 45267, USA.
[2]Departamento de Bioquímica, Universidade Federal de São Paulo, São Paulo, São Paulo, Brazil.
*These authors contributed equally to this work.

*Corresponding Email: tarsis.ferreira@gmail.com

Supplemental Figure 1: Ligplot plots for each peptide from averaged 100ns Molecular Dynamics simulations.

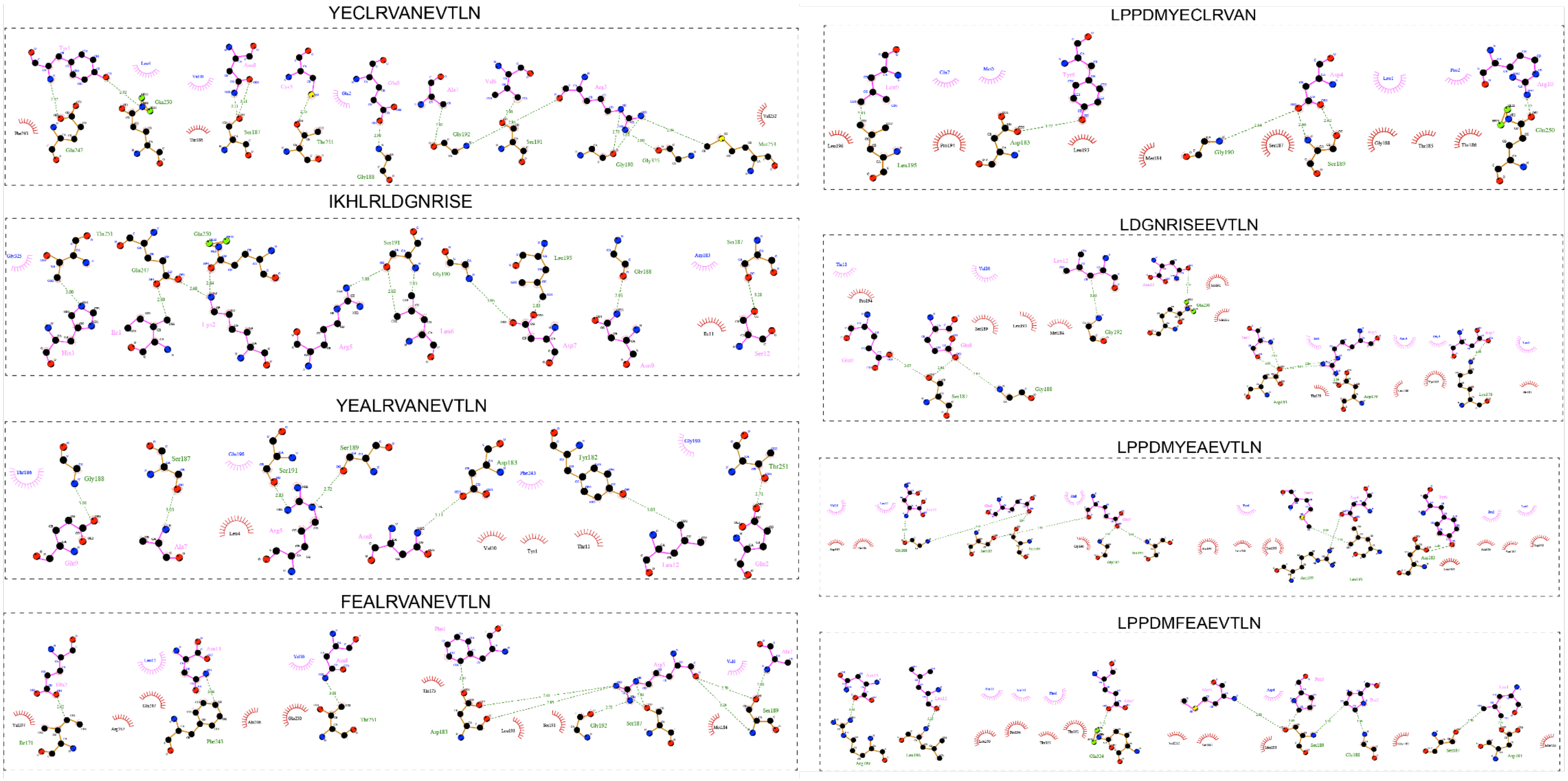

Supplemental Figure 2: Representative measurement of migration of epithelium sheet experiments. Lum-/- mice were subjected to corneal epithelium debridement and treated with LumC peptides as described in Figure 4.

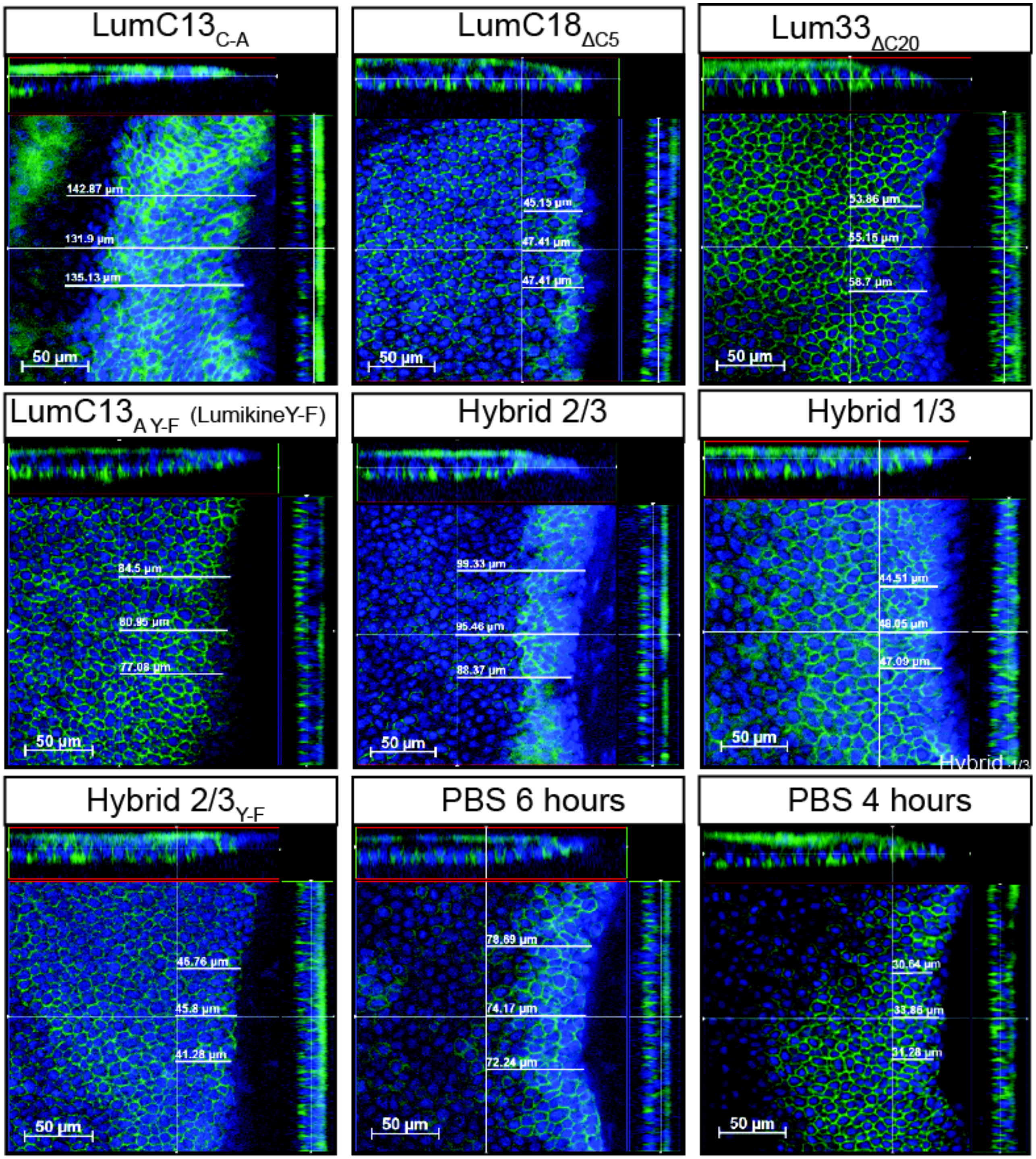

Supplemental Table 1: Haddock scores

	Cluster[a]	Haddock score (kcal mol-1)[b]	N_c	RMSD-E_{min} (Å)[d]	E_{VDW} (kcal mol^{-1})[e]	E_{elec} (kcal mol^{-1})[f]	E_{desolv} (kcal mol^{-1})[g]	E_{AIR} (kcal mol^{-1})[h]	BSA (Å^2)[i]
LumC13	1	-83.6 ± 7.3	82	2.8 ± 0.1	-40.9 ± 6.3	-436.1 ± 24.2	42.4 ± 3.6	21.8 ± 15.74	1290.8 ± 109.1
	3	-81.1 ± 10.3	14	0.6 ± 0.4	-38.9 ± 3.1	-368.9 ± 37.5	49.5 ± 4.5	21.0 ± 14.81	1196.7 ± 53.5
	4	-79.8 ± 13.3	12	4.4 ± 0.2	-38.7 ± 3.2	341.2 ± 67.0	45.1 ± 13.2	21.1 ± 16.30	1321.3 ± 72.3
	5	-74.4 ± 5.9	11	3.7 ± 0.3	-42.9 ± 6.3	-336.7 ± 38.3	54.5 ± 6.3	14.1 ± 22.75	1246.6 ± 61.8
	11	-69.9 ± 6.7	5	4.7 ± 0.2	-41.7 ± 8.8	-298.6 ± 25.9	49.7 ± 7.7	17.9 ± 16.09	1265.9 ± 121.5
	10	-66.4 ± 13.3	6	4.0 ± 0.3	-33.7 ± 6.7	-246.8 ± 54.6	56.1 ± 5.1	6.6 ± 6.46	1083.0 ± 275.3
	2	-65.7 ± 5.6	18	2.2 ± 0.4	-30.9 ± 4.2	-231.6 ± 26.5	53.1 ± 3.5	44.4 ± 43.97	1237.8 ± 77.0
	6	-63.7 ± 5.1	11	4.3 ± 0.1	-24.1 ± 3.1	-222.0 ± 52.9	51.5 ± 6.6	6.9 ± 4.41	6.9 ± 4.41
	7	-54.0 ± 10.2	9	1.6 ± 0.6	-30.9 ± 5.2	-211.5 ± 54.4	55.5 ± 8.5	16.0 ± 19.99	953.6 ± 188.1
	8	-46.6 ± 3.8	7	3.5 ± 0.1	-25.6 ± 5.0	-206.2 ± 25.5	52.6 ± 5.3	8.9 ± 5.58	910.6 ± 80.4
LumC30ΔC20	5	-77.3 ± 11.4	10	0.6 ± 0.3	-45.5 ± 8.0	-231.0 ± 23.3	31.1 ± 2.8	32.9 ± 9.99	1375.6 ± 140.2
	1	-71.7 ± 6.1	74	1.1 ± 0.3	-37.0 ± 4.6	-243.4 ± 20.4	29.9 ± 4.9	39.8 ± 15.45	1213.5 ± 98.9
	2	-67.9 ± 1.5	13	4.9 ± 0.1	-35.9 ± 0.9	-207.9 ± 34.7	26.2 ± 8.6	34.4 ± 19.07	1135.8 ± 57.8
	3	-60.7 ± 3.7	13	4.3 ± 0.0	-40.8 ± 5.4	-145.2 ± 31.1	27.8 ± 6.4	12.5 ± 11.06	1181.5 ± 121.8
	7	-54.3 ± 10.8	6	1.7 ± 0.1	-33.0 ± 5.2	-191.1 ± 45.1	33.3 ± 8.8	36.0 ± 22.55	1082.6 ± 158.5
	4	-51.7 ± 7.0	12	2.0 ± 0.1	-43.0 ± 5.4	-53.4 ± 8.4	20.9 ± 4.8	11.4 ± 12.52	1093.5 ± 88.5
	8	-50.2 ± 5.3	6	4.2 ± 0.0	-41.8 ± 4.0	-66.8 ± 37.5	31.2 ± 5.6	17.1 ± 12.71	1229.5 ± 42.0
	6	-49.7 ± 4.8	6	1.2 ± 0.2	-30.7 ± 3.5	-134.6 ± 32.5	34.4 ± 3.2	35.4 ± 13.26	1053.0 ± 117.8
	10	-40.6 ± 11.6	4	1.4 ± 0.2	-23.2 ± 11.7	-146.0 ± 24.9	39.7 ± 8.6	21.0 ± 17.44	837.0 ± 308.1
	9	-20.0 ± 12.4	4	4.9 ± 0.1	-26.3 ± 5.6	-114.8 ± 23.1	37.0 ± 13.9	22.9 ± 30.62	780.8 ± 105.0
LumC13C-A	5	-53.8 ± 7.1	7	4.3 ± 0.1	-43.4 ± 3.5	-153.0 ± 29.2	36.0 ± 7.2	42.1 ± 27.24	1384.9 ± 79.8
	1	-52.4 ± 4.3	67	4.1 ± 0.1	-31.6 ± 8.5	-223.4 ± 75.5	42.8 ± 12.3	11.0 ± 12.56	1288.0 ± 116.1
	2	-52.2 ± 6.2	22	1.2 ± 0.1	-41.8 ± 2.2	-177.7 ± 10.9	44.1 ± 5.3	9.8 ± 13.06	1257.2 ± 73.8
	3	-36.8 ± 4.8	9	4.2 ± 0.1	-30.0 ± 7.3	-148.8 ± 86.7	39.8 ± 7.9	32.0 ± 17.75	1111.3 ± 54.9

	10	-36.3 ± 19.8	5	4.6 ± 0.1	-25.0 ± 10.3	-179.1 ± 64.4	43.5 ± 7.1	9.7 ± 12.48	786.9 ± 121.4
	4	-31.4 ± 9.0	8	4.1 ± 0.1	-35.4 ± 4.7	-81.2 ± 9.0	35.0 ± 5.4	53.0 ± 16.07	1137.3 ± 59.7
	12	-30.2 ± 27.7	4	0.9 ± 0.6	-36.2 ± 12.7	-111.1 ± 37.6	46.1 ± 10.4	21.6 ± 18.00	1063.5 ± 289.6
	9	-27.1 ± 9.6	5	3.0 ± 0.3	-27.8 ± 5.3	-126.0 ± 60.5	42.4 ± 4.6	34.5 ± 18.50	985.9 ± 96.5
	7	-26.2 ± 10.0	5	4.1 ± 0.1	-25.6 ± 6.3	-177.1 ± 49.8	50.9 ± 4.9	38.9 ± 23.42	1094.3 ± 144.0
	6	-24.7 ± 9.2	5	3.5 ± 0.2	-30.8 ± 4.4	-118.2 ± 56.7	45.2 ± 6.0	45.0 ± 27.23	969.7 ± 150.7
LumC13AY-F	1	-84.8 ± 7.0	52	1.1 ± 0.7	-49.8 ± 7.1	-313.1 ± 40.4	23.8 ± 16.6	37.4 ± 28.55	1574.4 ± 138.3
	4	-72.3 ± 4.4	20	3.5 ± 0.0	-55.7 ± 5.1	-279.1 ± 23.2	37.6 ± 4.1	16.6 ± 24.55	1685.6 ± 41.9
	3	-58.0 ± 3.8	27	1.1 ± 0.4	-37.7 ± 6.1	-327.7 ± 17.1	44.1 ± 10.7	12.7 ± 13.73	1355.1 ± 125.7
	9	-55.7 ± 21.8	6	1.1 ± 0.5	-38.1 ± 3.6	-272.0 ± 97.4	36.3 ± 3.0	5.7 ± 3.63	1375.6 ± 159.1
	2	-53.3 ± 2.5	32	4.6 ± 0.1	-28.0 ± 9.3	-311.6 ± 85.6	34.4 ± 12.6	26.4 ± 20.09	1121.5 ± 51.1
	6	-41.4 ± 6.9	10	2.6 ± 0.1	-38.5 ± 4.6	-241.9 ± 73.4	41.4 ± 13.1	41.6 ± 11.45	1340.6 ± 146.7
	8	-38.7 ± 9.1	7	4.0 ± 0.1	-31.7 ± 3.0	-246.9 ± 56.5	39.7 ± 9.0	26.7 ± 24.95	1059.0 ± 34.6
	5	-37.9 ± 7.2	17	3.6 ± 0.1	-38.1 ± 3.2	-170.9 ± 48.6	33.6 ± 8.3	8.1 ± 10.90	1200.8 ± 98.1
	7	-34.4 ± 4.6	8	1.8 ± 0.3	-37.1 ± 1.6	-140.8 ± 21.7	30.5 ± 3.4	3.8 ± 1.83	1170.7 ± 141.1
LumC18ΔC5	3	-79.5 ± 1.7	25	0.5 ± 0.3	-48.2 ± 7.2	-453.9 ± 27.8	42.4 ± 6.1	10.4 ± 5.86	1450.3 ± 71.0
	9	-70.5 ± 15.0	4	2.9 ± 0.2	-47.7 ± 5.4	-441.8 ± 15.9	32.0 ± 7.4	35.9 ± 6.40	1474.0 ± 174.4
	2	-68.4 ± 5.7	36	3.1 ± 0.0	-48.4 ± 9.2	-455.7 ± 22.7	38.8 ± 3.5	23.5 ± 16.99	1517.9 ± 130.4
	5	-65.9 ± 3.7	9	4.7 ± 0.7	-37.5 ± 7.9	-390.2 ± 36.8	45.3 ± 5.3	43.6 ± 25.57	1169.4 ± 97.1
	1	-63.9 ± 3.9	52	3.6 ± 0.2	-41.5 ± 6.0	-290.9 ± 47.1	34.6 ± 2.2	12.0 ± 14.98	1080.8 ± 117.1
	7	-40.0 ± 5.7	5	2.3 ± 0.2	-32.4 ± 4.8	-266.4 ± 32.5	41.8 ± 3.3	29.0 ± 22.66	1106.4 ± 52.9
	4	--30.4 ± 1.6	13	2.6 ± 0.3	-27.0 ± 3.4	-201.5 ± 31.3	39.5 ± 4.6	34.1 ± 33.04	917.8 ± 51.4
	6	-29.1 ± 7.4	8	4.0 ± 0.2	-28.9 ± 4.8	-259.7 ± 30.3	34.6 ± 5.1	21.4 ± 30.83	781.6 ± 89.0
	2	-23.4 ± 7.8	4	4.1 ± 0.1	-39.0 ± 3.0	-245.8 ± 24.7	39.4 ± 7.6	53.7 ± 29.29	983.4 ± 52.7
	8	-3.2 ± 8.6	6	4.2 ± 0.1	-28.4 ± 3.9	-262.0 ± 45.4	37.3 ± 3.7	33.5 ± 33.41	886.7 ± 36.6
Hybrid 1/3	4	-55.8 ± 4.0	73	2.4 ± 0.2	-41.7 ± 9.0	-307.5 ± 57.5	46.6 ± 10.6	7.9 ± 3.80	1266.2 ± 138.5
	2	-53.7 ± 7.8	14	0.9 ± 0.6	-40.4 ± 7.2	-318.2 ± 54.8	49.4 ± 3.5	9.1 ± 11.73	1416.2 ± 26.6
	3	-43.4 ± 2.8	29	2.7 ± 0.0	-37.0 ± 2.0	-219.9 ± 34.8	37.0 ± 6.0	6.5 ± 4.59	1319.9 ± 57.6

	5	-38.3 ± 5.6	9	2.6 ± 0.3	-28.7 ± 4.1	-283.7 ± 27.0	46.4 ± 9.7	7.5 ± 10.10	1349.5 ± 64.9
	9	-36.0 ± 6.2	21	2.9 ± 0.0	-39.8 ± 5.9	-178.2 ± 30.5	36.6 ± 2.6	28.0 ± 15.21	1133.5 ± 45.2
	7	-30.3 ± 10.3	10	3.1 ± 0.2	-34.0 ± 4.3	-205.7 ± 39.3	42.1 ± 8.3	26.9 ± 25.29	1194.5 ± 82.5
	8	-15.5 ± 8.6	5	3.5 ± 0.3	-26.5 ± 4.6	-158.3 ± 87.5	40.3 ± 7.4	23.1 ± 15.66	1028.8 ± 67.8
	9	-7.1 ± 11.3	6	2.1 ± 0.2	-23.5 ± 2.4	-133.4 ± 88.2	37.9 ± 11.8	52.3 ± 17.58	861.5 ± 119.0
	10	-3.8 ± 3.7	6	3.4 ± 0.1	-23.6 ± 5.0	-102.1 ± 52.0	36.5 ± 9.3	37.4 ± 3.28	713.2 ± 25.6
Hybrid 2/3	1	-76.0 ± 1.6	137	0.4 ± 0.2	-55.2 ± 2.8	-384.7 ± 11.5	15.5 ± 3.3	6.5 ± 2.02	1441.9 ± 35.8
	2	-43.9 ± 5.1	45	2.9 ± 0.1	-42.3 ± 5.2	-118.0 ± 13.9	16.7 ± 5.7	53.0 ± 33.03	1231.0 ± 47.1
	4	-33.1 ± 5.8	5	3.0 ± 0.1	-33.4 ± 9.4	-194.5 ± 57.2	38.0 ± 9.3	12.3 ± 12.21	1215.6 ± 113.2
	3	-18.4 ± 9.2	8	3.6 ± 0.1	-27.4 ± 5.5	-157.4 ± 7.4	38.7 ± 3.6	17.3 ± 13.12	1040.8 ± 50.8
	5	-17.8 ± 14.1	4	4.1 ± 0.0	-38.2 ± 10.1	-89.7 ± 27.5	26.3 ± 6.4	120.5 ± 27.40	1071.3 ± 206.2
Hybrid 2/3Y-F	1	-73.7 ± 2.1	99	0.5 ± 0.3	-45.3 ± 5.0	-270.4 ± 11.8	24.8 ± 3.9	9.1 ± 2.89	1284.2 ± 24.2
	3	-53.8 ± 8.7	12	2.6 ± 0.0	-39.7 ± 7.1	-126.9 ± 24.4	9.7 ± 9.9	15.8 ± 15.18	1324.2 ± 124.0
	2	-47.5+-3.6	16	2.9 ± 0.2	-41.0 ± 8.5	-140.6 ± 34.0	20.5 ± 5.0	10.4 ± 2.37	1294.3 ± 166.5
	4	-40.1 ± 7.0	10	3.4 ± 0.0	-39.6 ± 3.5	-200.8 ± 21.2	36.8 ± 6.5	27.9 ± 9.84	1288.3 ± 60.7
	5	-28.4 ± 5.1	8	2.8 ± 0.1	-35.9 ± 4.8	-83.5 ± 24.6	23.3 ± 2.8	8.4 ± 5.30	1074.6 ± 86.2
	6	-7.6 ± 13.7	4	3.5 ± 0.1	-28.6 ± 3.4	-48.1 ± 29.5	28.6 ± 7.7	20.4 ± 13.05	899.0 ± 26.7
	7	-7.2 ± 7.6	4	2.4 ± 0.2	-29.9 ± 8.2	-84.8 ± 37.3	35.4 ± 11.5	41.5 ± 25.79	1253 ± 63
